# Automatic identification of postural transitions using a single inertial measurement unit and dynamic time warping: a pilot study in healthy individuals and people with Parkinson’s disease

**DOI:** 10.3389/fbioe.2026.1787510

**Published:** 2026-07-27

**Authors:** Cinzia Amici, Roberto Bussola, Joel Pollet, Massimiliano Gobbo, Riccardo Buraschi

**Affiliations:** 1 Department of Mechanical and Industrial Engineering, University of Brescia, Brescia, Italy; 2 Department of Clinical and Experimental Sciences, University of Brescia, Brescia, Italy; 3 IRCCS Fondazione Don Carlo Gnocchi, Milan, Italy

**Keywords:** assessment, dynamic time warping, inertial measurement unit (IMU), Parkinson’s disease, postural transition

## Abstract

**Introduction:**

Postural transitions (PTs) are crucial daily movements often impaired in neurological conditions, impacting autonomy and fall risk. Wearable inertial measurement units (IMUs) enable objective assessment of PTs, but robust algorithms for automatic identification remain limited.

**Methods:**

This pilot study used Dynamic Time Warping (DTW), a time-series alignment method that is robust to temporal variations, to automatically identify PTs in healthy subjects (HS) and subjects with Parkinson's Disease (SwPD). For this purpose, 10 participants (5 HS and 5 SwPD) performed 5 postural transition tasks (sit-to-stand, stand-to-sit, supine-to-sit, sit-to-supine, and roll) using a sternum-mounted IMU. Reference PT patterns were generated from previously collected acceleration data representing optimally executed transitions. The DTW algorithm classified each detected candidate transition by minimizing its distance from predefined reference patterns. Classification performance indexes were statistically compared between groups.

**Results:**

Within this pilot dataset, the DTW algorithm correctly identified 118/118 postural-transition signals in HS and 136/147 signals in SwPD. Misclassifications in SwPD primarily affected sit-to-stand (31%) and stand-to-sit (19%) transitions.

**Discussion:**

These preliminary findings support the feasibility of a DTW-based approach for postural-transition identification, although larger independent validation studies are required. Future work should prioritize real-time deployment and larger validation cohorts.

## 1 Introduction

POSTURAL transitions (PTs) are literally the transition from one specific posture to another, e.g., from sitting to standing, or from lying supine to side lying. PTs are common activities essential for every person to perform across different daily living settings. These activities play an important role in the clinical context, as subjects with disabling conditions, like Subjects with Parkinson’s disease (SwPD), commonly have difficulty executing PTs. Such conditions imply a reduction in the autonomous execution of the Activities of Daily Living (ADL) and involve movement deficits that have a significant impact on pivotal categories of the International Classification of Functioning, Disability and Health ICF (Stucki, 2005), namely, body structures and function, activities, and participation.

Among the motor symptoms of SwPD, a disabling condition is represented by the difficulties in executing PTs. The combination of balance impairments, plastic muscle hypertonia, bradykinesia, and other symptoms heavily affects the ability of SwPD to execute PTs. A clear example is the sit-to-stand transition in which SwPD have to try several times to stand up before executing a successful PT. Rehabilitation and physiotherapy help SwPD in improving PTs’ execution. Indeed, in rehabilitation medicine, attention to the ability to perform PTs safely and in a time congruent with the concept of autonomy is a priority, and monitoring and evaluating them yield multiple benefits for health and medical rehabilitation. In a clinical setting, a PT’s evaluation could serve as an objective and functional outcome measure for therapeutic intervention or as valuable feedback to enhance patients’ adherence and compliance with the proposed treatment.

Similarly, in home- or community-based settings, the ability to monitor PTs enables accurate assessment over time following the rehabilitation course. In particular, differences between patients’ movement capacity (what a person can do in a standardized, controlled environment) and movement performance (what a person actually does in their daily environment) can be detected. Nonetheless, health surveillance can be implemented to identify motor behaviors that warrant attention in the frailest patients (e.g., unsafe motor performance, increased fall risk, low physical activity, and sedentary lifestyle) and to detect adverse events such as falls.

Inertial Measurement Units (IMUs) are a valuable technology for assessing motor activities (Bolam et al., 2021; Rodríguez-Martín et al., 2013) and have been extensively used in clinical contexts. Specifically, Amici 2024 et al. (Amici et al., 2024) proposed applying IMU-based kinematic analysis to PTs, suitable for rehabilitation setting assessment. IMU devices are wearable and reasonably comfortable, usually equipped with long-lasting batteries and data storage capabilities, or with continuous data transmission features, all of which have also made them suitable for remote monitoring of human movement. The ability to remotely monitor PTs is of great value for the clinical and health surveillance implications previously mentioned. When monitoring PTs, the first step is to ensure PT recognition, i.e., the ability to identify and discern a PT within the totality of movements performed by the monitored subject. Wearable activity monitors and single-sensor inertial systems have been used to detect posture-related events and quantify sit-to-stand and stand-to-sit transitions in laboratory, home-like, and free-living conditions (Adamowicz et al., 2020; Zijlstra et al., 2012; Buraschi et al., 2022). Nevertheless, most available approaches remain focused on selected transitions, particularly sit-to-stand and stand-to-sit, and are commonly linked to specific sensor placements or device-specific processing procedures.

Various techniques can be employed to analyze the signals detected from IMUs. These methodologies are mostly applied to acceleration signals or angular velocities; however, they are technically applicable to signals of other types. Conventionally, the prevailing techniques can be categorized into three distinct classifications: time-domain analyses (e.g., based on threshold detection, identification of relevant events like picks, over/undershoots, minima or maxima, zero-crossing detection), frequency-domain analyses (e.g., Fast Fourier Transform, FFT), and time-frequency analyses (e.g., Wavelet transform). Presently, these techniques may also be applied to extract features and used in combination with machine/deep learning techniques or artificial intelligence methods to implement autonomous classification strategies. Furthermore, in recent years, machine learning and deep learning methods have become increasingly prominent in wearable-sensor-based human activity recognition. Compared with conventional approaches based on predefined thresholds or handcrafted features, these methods can automatically learn complex temporal patterns from raw or minimally processed inertial data and may achieve high classification performance across multiple activities. Recent reviews have highlighted the rapid development of CNN-, LSTM-, Transformer-, and ensemble-based models for HAR using smartphones, smartwatches, and wearable sensors.

Each approach presents strong points and drawbacks. Threshold-based approaches are attractive because they are relatively simple, interpretable, and potentially suitable for online implementation. However, their performance may depend on event definitions, window length, sensor location, and thresholds optimized on a specific dataset or population, which may limit transferability when movement execution differs across subjects or clinical and experimental conditions. Previous studies have therefore emphasized the importance of validating such algorithms across sensor locations, populations, and real-life or home-like conditions (Ding et al., 2008; Wang et al., 2016). Frequency- and time-frequency-domain approaches can provide valuable information on the structure of movement signals, but they generally require sufficiently informative signal windows and are often applied in offline or post-processing contexts. In contrast, template-based methods such as Dynamic Time Warping (DTW) are specifically designed to compare temporal sequences that may differ in duration, timing, or execution speed (Yang et al., 2019; de Azevedo et al., 2016; Mangalam et al., 2024). Data-driven models (machine learning and deep learning approaches) also have important limitations in early-stage clinical applications: they generally require large, well-annotated, and representative datasets, and their performance may be strongly influenced by sensor placement, population characteristics, activity definitions, and validation strategy (Sm et al., 2025; Qureshi et al., 2025).

When working with IMU data for movement recognition, the primary challenge is that the same movement can be performed at different speeds or with different durations by different individuals or even by the same individual at different times. This temporal variation makes it difficult to directly compare raw sensor data with a reference profile using traditional distance metrics, such as Euclidean distance, because they assume the sequences have the same length and alignment. DTW addresses this issue by allowing for non-linear mappings between the two sequences, effectively “warping” the time dimension of one of the profiles to find an optimal alignment that minimizes the overall distance between them. This is particularly useful for movement recognition because it can account for variations in speed, duration, and timing while still capturing the essential patterns and shapes of the data. The DTW algorithm works by constructing a cost matrix, where each element represents the distance between two points from the two sequences being compared. It then finds the optimal warping path through this matrix, which represents the best alignment between the sequences and minimizes the overall distance or dissimilarity between them.

However, a fundamental distinction must be made between techniques that analyze signals offline and in real time, as for the latter, the running time of the code must be taken into account, as well as the overall computational burden, so that the software must be optimized to provide timely results, in line with the needs and expectations of the program. This heavily affects, for instance, the types of algorithms that can be implemented, as only algorithms that provide results within a certain time limit should be used, and those algorithms must be sustainable in terms of computational burden (e.g., the number and types of operations allowed by the hardware in use).

Consequently, developing a specific algorithm to detect and identify PTs is essential for this kind of assessment. In this context, adopting a well-known method like DTW is novel in the understudied field of PTs and could significantly improve the ability to study these important activities in different settings.

In this study, we propose a DTW-based application for IMU data to automatically identify PTs in populations of healthy subjects (HS) and subjects with Parkinson’s disease (SwPD), and to apply it with a vectorial approach to more than two signals at a time. The study provides a preliminary offline proof-of-concept of a DTW-based approach for PT identification, with an algorithmic structure that may be further developed and specifically validated for future online implementation.

## 2 Materials and methods

### 2.1 Study design

This cross-sectional observational study was approved by a local Ethical Committee (Ethical Committee of Fondazione Don Carlo Gnocchi, protocol number: CE_FdG_1642020) and conducted in accordance with the Helsinki Declaration.

### 2.2 Population

We recruited a convenience sample of subjects with Parkinson’s disease (SwPD) and age-matched healthy volunteers (HS) from tertiary referral rehabilitation research facilities. Eligibility criteria adopted were i) adults, ii) both sexes, iii) absence of past or present pathologies that could affect movement presently for the healthy subset, and iv) diagnosis of Parkinson’s disease for the pathological subset. Written informed consent was obtained from all participants. SwPD were acquired in the medication ON/IN phase, had Hoehn and Yahr stages between 3 and 4, and had preserved global cognitive status as indicated by MMSE >24. Written informed consent was obtained from all participants.

### 2.3 Experimental setup

The research was conducted at the MoRe–R2 Laboratory of IRCCS Fondazione Don Carlo Gnocchi in Rovato, Italy. Subjects, both SwPD and HS, were asked to perform five tasks, three times each, on a standard physiotherapy bed, 46 cm high from the ground. A single IMU was attached to their bodies through a double-sided adhesive tape (precisely at the level of the xiphoid process of the sternum), as proposed in previous studies (Amici et al., 2024; Anastasi et al., 2019). The tasks required were sit-to-stand, stand-to-sit, supine-to-sit, sit-to-supine, and roll. Each PT was repeated 3 times per subject; no instructions or constraints were provided to the subjects on how to execute the PT (Amici et al., 2024).

The IMU utilized for data capture was the G-Sensor device (BTS Bioengineering, Italy). This unit incorporates a triaxial accelerometer with selectable sensitivity levels (±2, ±4, ±8, and ±16 g), set to ±2 g for the acquisitions, and a triaxial magnetometer (13-bit resolution, ±1200 µT). Additionally, it includes a triaxial gyroscope with adjustable sensitivity settings (±250, ±500, ±1000, and ±2000° /s), set at ±2000° /s in the current case, along with a GPS receiver capable of achieving a positional accuracy of 2.5 m at up to 5 Hz or 3.0 m at up to 10 Hz. The device itself measures 70.0 × 40.0 × 18.0 mm in volume. This inertial unit provides data on acceleration and angular velocity at up to 200 frames per second. Furthermore, sensor fusion technology could determine both orientation and position. In this study, data were collected at 100 Hz. The device communicated with a laptop via Bluetooth 3.0 (class 1.5).

### 2.4 Data processing

The data from the triaxial accelerometer of the IMU were exported and elaborated in the Delphi environment with custom code. In the present study, PT detection and classification were performed offline on previously acquired IMU signals. Although the algorithmic structure was conceived to be compatible with future online implementation, no fully real-time acquisition-and-classification system was validated in this pilot study.

Preliminarily, for each PT, a mean profile of each acceleration component was estimated, which represents a qualitative pseudo-normal pattern to be used as a reference signal for comparison. Each PT can be treated as a distinct class in a classification problem, and the qualitative reference patterns are the corresponding features for each class. In particular, those reference patterns were computed from a previously acquired dataset of healthy subjects repeating five times a well-performed PT, according to two physiotherapists with at least 10 years of experience in movement analysis and neurorehabilitation (J.P., R.B.). Consequently, the mean of the five acquisitions, each resampled to a fixed length of 100 samples (corresponding to 1 s at a sampling rate of 100 Hz), was used to determine the reference pattern for each PT. As the evaluation is performed for each acceleration component, each PT is described with three profiles.

**FIGURE 1:**
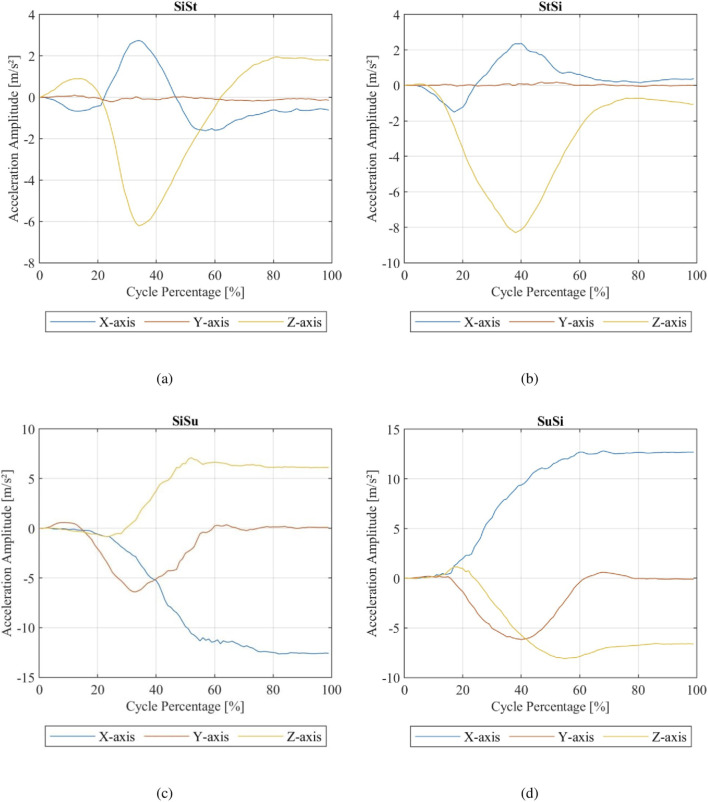
Reference patterns of the sit-to-stand **(a)**, stand-to-sit **(b)**, sit-to-supine **(c)**, and supine-to-sit **(d)** PT. In red line, the acceleration signal along the X-axis, in green, the acceleration along the Y-axis, and in blue, the acceleration along the Z-axis.

**FIGURE 2:**
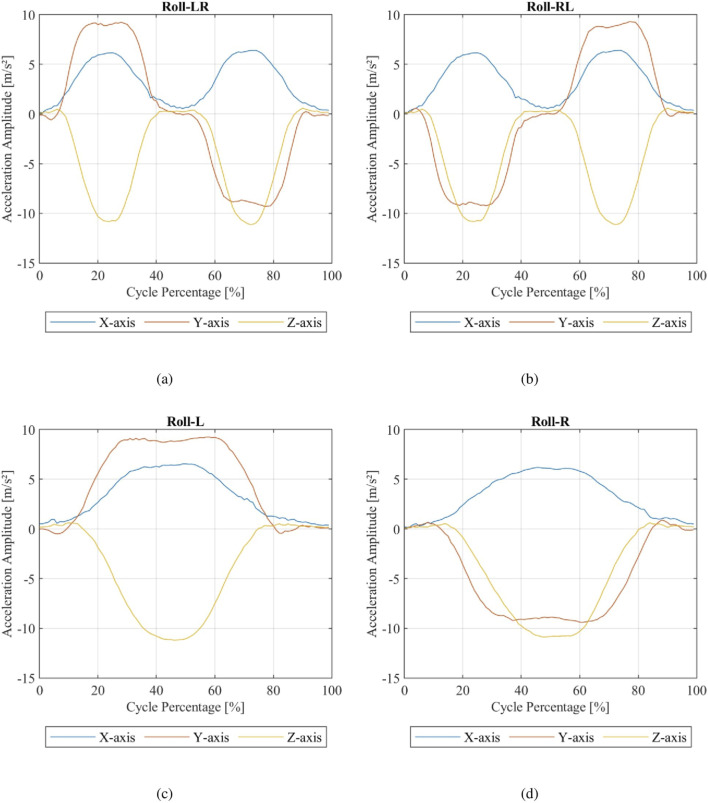
Reference patterns of Roll performed according to the current protocol, towards left and then towards right Roll-LR **(a)**, of the Roll performed in the opposite order Roll-RL **(b)**, of the partial Roll, performed rolling onto the left shoulder and back to central position Roll-L **(c)**, and of the partial Roll, performed rolling onto the right shoulder and back to central position Roll-R **(d)** PT. In red line, the acceleration signal along the X-axis, in green, the acceleration along the Y-axis, and in blue, the acceleration along the Z-axis.

**FIGURE 3:**
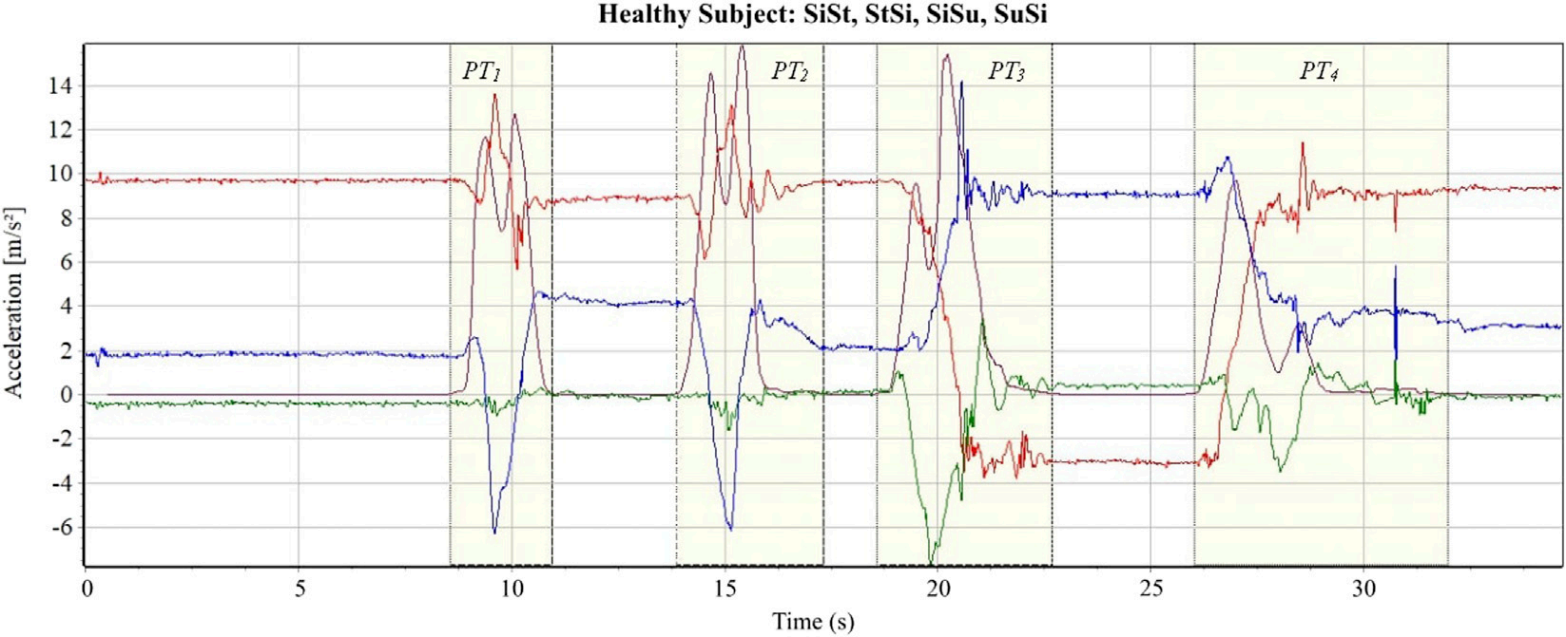
Example of signals acquired for a sit-to-stand, stand-to-sit, sit-to-supine, and supine-to-sit for a healthy subject. In red line, the acceleration signal along the X-axis, in green, the acceleration along the Y-axis, and in blue, the acceleration along the Z-axis. In magenta, the norm of the three signals. The yellow areas indicate the detected PTs between the events at *t*_*start*_ and *t*_*stop*_.

**FIGURE 4:**
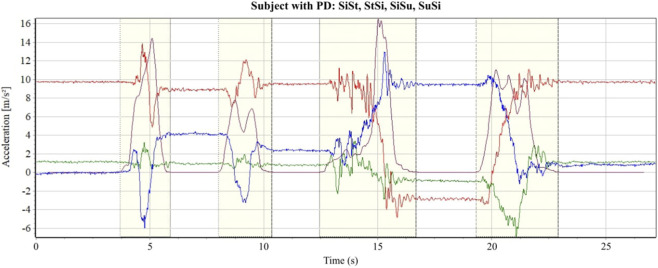
Example of signals acquired for a sit-to-stand, stand-to-sit, sit-to-supine, and supine-to-sit for a subject with Parkinson’s disease. In red line the acceleration signal along the X-axis, in green the acceleration along the Y-axis, and in blue the acceleration along the Z-axis. In magenta is the sliding-window norm of the variances of the three signals. The yellow areas show the detected PTs between events of *t*_*start*_ and *t*_*stop*_.

**FIGURE 5:**
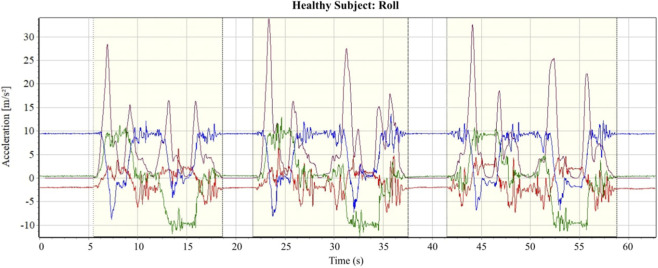
Example of signals acquired for three Roll PTs, for a healthy subject. In red line the acceleration signal along the X-axis, in green the acceleration along the Y-axis, and in blue the acceleration along the Z-axis. In magenta, the sliding-window norm of the variances of the three signals. The yellow areas show the detected PTs between events of *t*_*start*_ and *t*_*stop*_.

**FIGURE 6:**
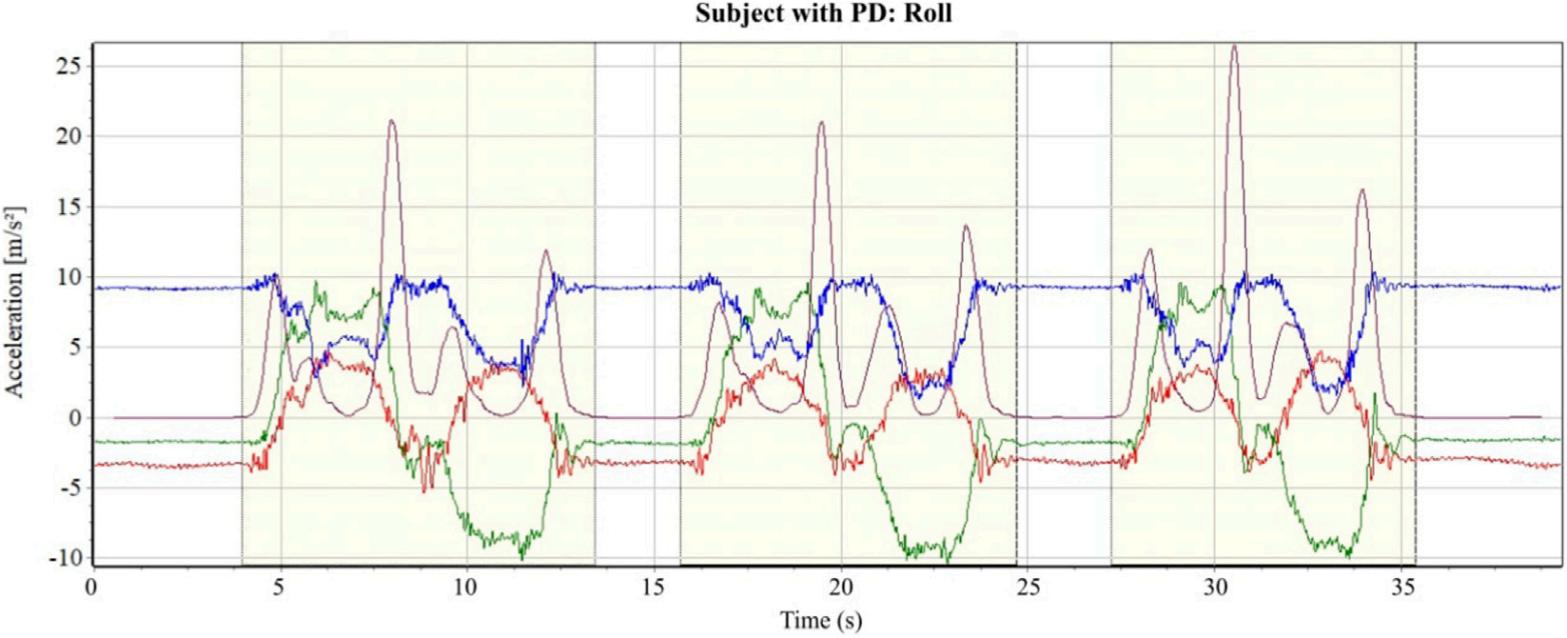
Example of signals acquired for three Roll PTs, for a subject with Parkinson’s disease. In red line the acceleration signal along the X-axis, in green the acceleration along the Y-axis, and in blue the acceleration along the Z-axis. In magenta, the sliding-window norm of the variances of the three signals. The yellow areas indicate the detected PTs between the events at *t*_*start*_ and *t*_*stop*_.

Special attention was given to the Roll PT, which in the applied acquisition protocol was defined as a rotation first to the left side, then to the right shoulder, and finally back to the central supine condition, with continuous movement by the subject. In real life, this PT may be performed in a different order or just partially, and all these options were evaluated as independent potential Roll patterns for the identification algorithm. Four independent reference patterns were therefore considered just for this PT, all derived from the reference pattern of the Roll PT performed according to the applied protocol, as part of the original pattern for the sub-tasks, or as their proper composition for the task in the opposite order. Figures 1, 2 depicts the final set of reference patterns used as classification features, and Figures 3–6 show examples of the actual signals for HS and SwPD during the different PTs.

In detail, the set of reference patterns is made up of ℛ = {**R**_c_ | c = 1,…,8}, where each of the eight templates **R**_c_ = [**r**_***x*** c_, **r**_***y*** c_, **r**_***z*** c_]^T^ is a matrix of 3 × *n* elements, with *n = *100. Each row of this matrix contains a vector of *n* samples for each of the *x*, *y*, and *z* components of the acceleration profile of the c-*th* PT. This profile is the average of five offset-cleaned acquisitions for that posture. Naturally, a higher number of template transitions than the five considered will provide more representative patterns, and from this point of view, it is conceivable to develop self-adaptive algorithms that update the templates whenever a pattern is recognized.

**FIGURE 7:**
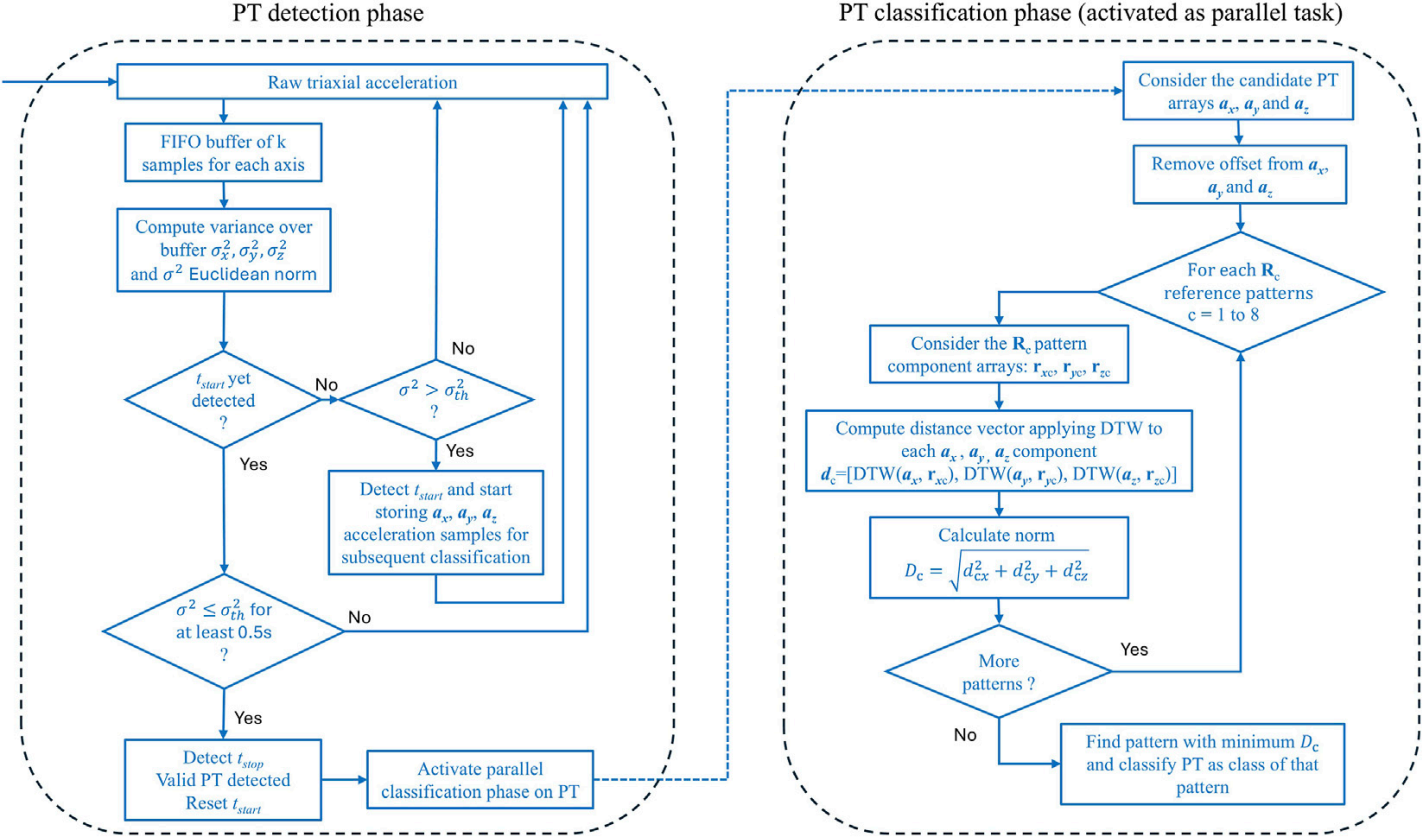
Flowchart reporting the algorithm pipeline.

Regarding the signal to be classified, the algorithm was structured into two phases: PT detection and PT classification, as shown in the simplified flowchart in Figure 7. In the present offline implementation, the first phase analyzed previously acquired acceleration signals to identify a window of samples that could represent a potential PT. In the second phase, classification was performed on the detected window based on the reference patterns ℛ.

In the PT detection phase, the acceleration components acquired by the IMU are considered to detect events representing the start and stop of a PT. For this purpose, a detection strategy was defined: for each axis (*x*, *y*, *z*), the last *k =* 50 acceleration samples (that is, 0.5 s) are stored in a FIFO buffer, thus representing a sliding window on the signal. The variance of each axis over this buffer is computed, yielding ${\sigma_{x}^{2},\sigma }_{y}^{2}{,\sigma }_{z}^{2}$ . The Euclidean norm of the three variances, i.e., $\sigma^{2}=\sqrt{\sigma_{x}^{4}+\sigma_{y}^{4}+\sigma_{z}^{4}}$ , is then compared with a threshold value of $\sigma_{th}^{2}=0.05$ m^2^/s^4^. For example, in Figures 3–6, each point of the magenta curves represents the sliding-window norm of the variances of the three signals. The first and last *k*/2 points are absent because the window has a width of k and the value is assigned to the window’s center. When this norm exceeds the threshold, the instant at the center of the buffer is detected as the initial *t*_*start*_ event of a potential PT, and acceleration samples begin to be stored for further manipulation; after *t*_*start*_ event is detected, when the norm falls stably below the threshold for at least 0.5 s, the instant at the center of the sliding window is set as a potential final instant *t*_*stop*_ of the PT. Now, the storage of acceleration samples ends up being subsequently processed in the classification phase. This combination (norm of variances per axis over the sliding window) was chosen over other quantities (e.g., variance of the norm of the acceleration components, mean of the norm, maximum of the components, etc.) because it preserves the independent contribution of each axis, is sensitive to changes in any direction, removes the gravitational offset, rejects slow drifts, and responds selectively to local signal fluctuations indicating movement onset/offset. Additional controls are then implemented on the potential events identified as a minimum acceptable number of elements (50 samples, i.e. 0.5s) for the start-stop interval, to limit the risk of false detections. The chosen threshold (0.05 m^2^/s^4^), as well as the width of the sliding window (50 samples), was determined empirically by repeating the tests on many acquisitions varying the parameters by ±20% and verifying a detection rate >95%. The computation time for a 50-sample window, on a standard PC equipped with an Intel(R) Core(TM) Ultra 7 258V (2.20 GHz) processor and Windows 11 Home operating system, is on the order of a few hundred microseconds, which allows it to be repeated during acquisition of new samples without overloading the system. This allows a parallel task dedicated to DTW processing to be kept active in case a potential PT is detected.

Once a potential PT is identified, the PT classification strategy is applied. The acceleration components in the interval [*t*_*start*_, *t*_*stop*_] are cleared of the offset by subtracting the value of their first sample and are represented by three vectors: ***a***_***x***_ ***=*** (*a*_*x1*_, *a*_*x2*_, …, *a*_*xm*_) for the *x*-component, ***a***_***y***_ ***=*** (*a*_*y1*_, *a*_*y2*_, … , *a*_*ym*_) and ***a***_***z***_ ***=*** (*a*_*z1*_, *a*_*z2*_, … , *a*_*zm*_) for the *y* and *z* components, where *m* denotes the number of samples that make up the detected window [*t*_*start*_, *t*_*stop*_]. For each reference pattern **R**_c_ = [**r**_***x*** c_, **r**_***y*** c_, **r**_***z*** c_]^T^, the DTW algorithm is applied to the acceleration vector [***a***_***x***_, ***a***_***y***_, ***a***_***z***_]^T^, component by component, obtaining the distance vector ***d***_c_ = [*d*_c*x*_, *d*_c*y*_, *d*_c*z*_] as ***d***_c_ = [DTW (***a***_***x***_, **r**_***x*** c_), DTW (***a***_***y***_, **r**_***y*** c_), DTW (***a***_***z***_, **r**_***z*** c_)] of the potential detected PT from the c-*th* matrix template. The squared Euclidean distance–a standard choice in many studies on physiological signals–is adopted as a distance metric in DTW applied to each acceleration component. The norm $D_{c}=\sqrt{d_{cx}^{2}+d_{cy}^{2}+d_{cz}^{2}}$ of the evaluated distances ***d***_c_ is then computed, so the DTW-vector distances are quantified for all eight patterns, i.e., {*D*_c_ |c=1,…,8}, and the signal is assigned to the PT class that presents the minimum value of *D*_c_. Additional controls allow further checks on the classification result, such as a threshold on the maximum acceptable value of *D*_c_ for each PT. Furthermore, as originally conceived, the method is open to the recognition of further PTs once the reference patterns have been defined. As an example, Table 1 summarizes the results obtained from processing the signals shown in Figure 3. Each column corresponds to a detected PT. The first row reports *t*_*start*_ and *t*_*stop*_ values of the PT, while the second row gives its amplitude expressed as the number of samples. All remaining cells contain the *D*_*c*_ distance of the considered PT, computed with each reference pattern matrix **R**_c_∈ℛ. For every column, the minimum value is highlighted in bold; this identifies the row of the recognized PT class. As can be observed, the minimum values are generally much lower than the other entries in the same column, and they locate the expected PT class.

**TABLE 1:** Example of processed signal outcomes.

Detected PT		PT_1_	PT_2_	PT_3_	PT_4_
*t*_*start*_ [s] - *t*_*stop*_ [s]		8.55 - 10.92	13.87 - 17.3	18.56 - 22.66	26.01 - 31.93
PT width [samples]		237	343	410	592
Roll R-L	*D*_*1*_ [m^2^/s^4^]	3638.00	3203.76	47640.65	18745.83
Roll L-S	*D*_*2*_ [m^2^/s^4^]	3825.33	3754.28	47769.96	19013.10
Roll R	*D*_*3*_ [m^2^/s^4^]	3553.69	3575.40	49799.57	18958.92
Roll L	*D*_*4*_ [m^2^/s^4^]	3854.32	3816.66	48091.85	18163.88
SiSt	*D*_*5*_ [m^2^/s^4^]	**90.97**	925.66	29078.75	52729.10
StSi	*D*_*6*_ [m^2^/s^4^]	772.59	**133.05**	40775.11	49089.18
SiSu	*D*_*7*_ [m^2^/s^4^]	7873.86	16810.30	**311.03**	142774.95
SuSi	*D*_*8*_ [m^2^/s^4^]	16113.12	16388.64	109668.36	**353.74**

Bold values indicate the minimum DTW-vector distance (Dc) within each column and therefore identify the PT class assigned by the algorithm.

Regarding the DTW procedure, to minimize computational time, we did not apply any signal normalization before DTW, because the acceleration range was identical across all tests and the reference patterns. However, we will re-evaluate these aspects in future works to account for the greater variability expected in larger or more heterogeneous cohorts. A window constraint (Sakoe-Chiba band) of 30 samples was applied. This value was chosen based on two considerations: all reference patterns have a fixed length of 100 samples, and a sensitivity analysis was performed by computing the DTW distance for increasing window sizes (10, 15, 20, 25, 30, 45, 50). The distance was found to decrease markedly up to about 25 samples and then generally level off. Hence, 30 samples appear to provide a safety margin above the stabilization point. Computational tests on all eight patterns demonstrate that the DTW-vector processing takes ≈20 ms on average and never exceeds 30 ms for postural transitions of ∼4–5 s. This performance makes the algorithm possibly feasible for further real-time use, especially on real-time OS PCs. Using a custom Delphi 10.4 multitasking application on the described PC, the processing times are largely met even under Windows Home o. s.

### 2.5 Data analysis

The statistical analysis was conducted using RStudio version 4.4.0 (R Core Team, 2024). Given the pilot nature of the study and the very small sample, no confirmatory hypothesis testing or power-based sample-size calculation was planned. The identification rate of PTs in HS and SwPD, as well as across PTs, is reported using descriptive statistics and relative frequency analysis (absolute numbers and percentages). Because multiple trials were acquired from the same participants, signal-level identification rates were treated as descriptive outcomes only and should not be interpreted as estimates derived from independent observations. Therefore, percentages were not interpreted as inferential estimates derived from independent observations, and conventional signal-level confidence intervals were not reported to avoid underestimating uncertainty due to pseudo-replication.

## 3 Results

Ten subjects were recruited for this study, specifically 5 HS and 5 SwPD. Demographic characteristics are detailed in Table 2. A total of 265 signals have been processed, of which 118 were from tasks performed by HS and 147 were from tasks performed by SwPD. These signals represent repeated trials nested within 10 participants; therefore, the reported identification rates should be interpreted descriptively at the signal level. The DTW algorithm has identified 100% of PTs performed by HS and 93% of PTs performed by SwPD, as presented in Table 3. In particular, for SwPD, DTW showed an incorrect identification rate of 31% for the sit-to-stand transition and 19% for the stand-to-sit transition. At the same time, the remaining PTs were correctly identified, as shown in Table 4. The confusion matrix (Table 5) further describes the class-wise distribution of classification outcomes of the expected postural-transition classes against the classes assigned by the DTW algorithm. This analysis showed that misclassifications occurred only in the SwPD group and were restricted to sit-to-stand and stand-to-sit transitions. Specifically, 8 sit-to-stand signals were classified as stand-to-sit, whereas 3 stand-to-sit signals were classified as sit-to-stand. Conversely, roll, sit-to-supine, and supine-to-sit transitions were correctly classified in all analyzed signals.

**TABLE 2:** Characteristics of healthy subjects and subjects with Parkinson’s disease.

Characteristics	HS *N = 5* ^1^	SwPD *N = 5* ^1^	p-value
Sex	​	​	p>0.99^2^
Females	2/5 [40%]	2/5 [40%]	​
Males	3/5 [60%]	3/5 [60%]	​
Age	66.20 (8.56)	65.40 (8.96)	p>0.99^3^
BMI	25.54 (3.57)	21.67 (2.31)	p = 0.15^3^
Hoehn & Yahr	//	3 [3/5] 4 [2/5]	​

1 n/N [%]; Data for the second and third columns of Age and BMI are provided as Mean (SD); Hoehn & Yahr level [number of subjects/total population]; HS, healthy subjects; SwPD, subjects with Parkinson’s Disease.

2 Fisher’s exact test.

3 Wilcoxon rank sum test.

**TABLE 3:** Identification rate of postural transitions in healthy subjects and subjects with Parkinson’s Disease.

Group	HS, N = 118^1^	SwPD, N = 147^1^
Correctly identified	118 [100%]	136 [93%]
Not identified	0 [0%]	11 [7%]

1 n [%]; HS, healthy subjects; SwPD, subjects with Parkinson’s Disease.

**TABLE 4:** Identification rate by postural transition in subjects with Parkinson’s disease.

Task	N^1^	Correctly identified	Not identified
Roll R	21	21 [100%]	0 [100%]
Roll L	21	21 [100%]	0 [100%]
Roll L-R	21	21 [100%]	0 [100%]
SiSt	26	18 [69%]	8 [31%]
SiSu	21	21 [100%]	0 [100%]
StSi	16	13 [81%]	3 [19%]
SuSi	21	21 [100%]	0 [100%]

1 n [%]; R, rightward; L, leftward; SiSt, sit-to-stand; SiSu, sit-to-supine; StSi, stand-to-sit; SuSi, supine-to-sit.

**TABLE 5:** Confusion matrix of DTW-based postural-transition classification.

Actual PT class	DTW-assigned PT class								Total
​	Roll Dx-Sx	Roll Sx-Dx	Roll Dx	Roll Sx	SiSt	StSi	SiSu	SuSi	​
Roll Dx-Sx	**4**	0	0	0	0	0	0	0	**4**
Roll Sx-Dx	0	**39**	0	0	0	0	0	0	**39**
Roll Dx	0	0	**37**	0	0	0	0	0	**37**
Roll Sx	0	0	0	**37**	0	0	0	0	**37**
SiSt	0	0	0	0	**34**	8	0	0	**42**
StSi	0	0	0	0	3	**29**	0	0	**32**
SiSu	0	0	0	0	0	0	**37**	0	**37**
SuSi	0	0	0	0	0	0	0	**37**	**37**
Total	**4**	**39**	**37**	**37**	**37**	**37**	**37**	**37**	**265**

Values are reported as the number of signal-level observations. Rows indicate the actual postural-transition class, whereas columns indicate the class assigned by the DTW algorithm. Dx, rightward; Sx, leftward; SiSt, sit-to-stand; StSi, stand-to-sit; SiSu, sit-to-supine; SuSi, supine-to-sit. Bold values within the confusion matrix indicate correctly classified observations, for which the actual and DTW-assigned PT classes coincide; bold values in the ‘Total’ row and column indicate marginal totals.

## 4 Discussion

The proposed method introduces an algorithm that efficiently analyzes the three acceleration components captured by a single inertial measurement unit (IMU) to identify the presence and type of PTs, namely, the five movements: sit-to-stand, stand-to-sit, supine-to-sit, sit-to-supine, and roll. Since only the acceleration signals are necessary, the procedure could be ideally applied in a simplified setting, using a triaxial accelerometer instead of an IMU, as those included in smartphones.

Within this pilot dataset, the DTW-based procedure correctly identified all postural-transition signals in HS and most signals in SwPD. These results should be interpreted as preliminary within-sample identification rates rather than as evidence of robust classification performance. Indeed, the small number of participants, the repeated-trial structure of the dataset, the absence of an independent validation cohort, and the lack of cross-validation prevent firm conclusions about generalizability. Therefore, the present findings support the technical feasibility of the proposed approach, but not its validated robustness across populations, settings, or sensor configurations.

Misclassifications in SwPD were mainly observed during sit-to-stand and stand-to-sit transitions. These tasks are clinically relevant and may be affected by motor impairments in Parkinson’s disease, aligning with existing knowledge that anticipatory postural adjustments and transitional movements are commonly impaired in PD, often due to bradykinesia, axial rigidity, or freezing episodes (Mangalam et al., 2024; Janssen Daalen et al., 2025). However, the present study did not directly test associations between misclassification and specific clinical variables such as bradykinesia, rigidity, freezing, balance impairment, or disease severity. Therefore, this observation should be considered hypothesis-generating and should be investigated in future studies, including clinical severity measures and larger samples.

On the technical side, the proposed DTW implementation has two main distinctive features. First, the DTW procedure was applied component-wise to the three acceleration axes rather than after reducing the triaxial signal to a single scalar representation, such as the resultant acceleration magnitude. Specifically, for each detected postural-transition window, DTW was computed separately for the x-, y-, and z-acceleration components against the corresponding components of each reference pattern. The three resulting distances were then combined by computing their Euclidean norm, which provided the overall DTW-vector distance used for classification. This strategy was adopted to preserve axis-specific and directional information that may be attenuated or lost when the acceleration components are collapsed into a scalar signal. Second, the algorithm integrates an automatic detection step based on the sliding-window variance of the acceleration components, allowing the identification of the start and end of a potential postural transition before DTW-based classification.

A strength of this study is represented by the automatic identification of *t*_*start*_ and *t*_*stop*_ within the analyzed signal, which makes the system intrinsically autonomous, without requiring data fusion techniques to enable feature recognition. Additionally, preliminary results suggest that the algorithm’s processing time may be compatible with future online implementation. However, this aspect requires dedicated validation in a fully real-time acquisition-and-classification framework. On the other hand, accurately detecting the events t_*start*_ and t_*stop*_ is a non-negligible liability for the algorithm. Since the identification strategy is based on the correct detection of starting and ending conditions of the movement (*t*_*start*_ and *t*_*stop*_) and these values are selected by comparison with a threshold, a short calibration tailored to the single subject should be suggested when considering the future real-time application, although the currently adopted thresholds have been revealed to be quite robust for the current dataset. This is especially true in the case of pathological subjects, who can be heavily impaired in the execution of some of the PTs, or all of them. For this reason, collecting examples of PTs’ patterns as performed by the individual during a short preliminary calibration phase could facilitate their recognition and correct classification, even in cases of unconventional movement execution. In this sense, calibrating the system would allow fine-tuning of the algorithm, personalized to the needs of the individual patient.

These impairments impact patients’ safety during functional tasks such as standing, sitting, or rolling in bed, increasing the risk of falls and reducing autonomy in daily activities. Wearable IMUs, such as the single-sensor system used in this study, offer an opportunity to provide an objective, quantitative, and continuous assessment of motor behaviors in real-world settings and clinical trials (Regnault et al., 2019). Using an IMU can help gather extra information, such as assessing movement quality through analyzing angular velocity or its changes, but it is not strictly necessary for PTs’ identification. Also, this technology might support clinical judgment and improve personalized therapy planning by providing data-driven insights into patient performance in different settings (Regnault et al., 2019).

In line with this approach, in previous work, the authors proposed a set of six parameters, among which smoothness J, fluency Fl, jerk variation Δj, and total time TT of the execution, that act as rigorous indicators of the movement kinematics during a PT and allow task-independent descriptions of movement (Amici et al., 2024).

DTW does not require large training datasets and provides an interpretable distance-based classification procedure that may be useful for early-stage clinical applications and pilot datasets. This is particularly relevant because machine learning and deep learning approaches for wearable-sensor-based human activity recognition generally require sufficiently large, well-annotated, and representative datasets, together with appropriate validation strategies, to support generalizability (Sm et al., 2025; Qureshi et al., 2025). However, the performance of template-based methods depends strongly on the representativeness of the reference patterns, the quality of the detected start–stop window, and the similarity between the tested movement and the predefined templates. Consequently, atypical or compensatory strategies, sensor misplacement, and heterogeneous movement execution, especially in neurological populations, may reduce classification performance (Sczuka et al., 2021; Kale et al., 2012). Future studies should compare this DTW-based approach with data-driven classifiers using larger datasets, independent test sets, and clinically heterogeneous populations.

Despite its promising results, this study has limitations. First, the sample size was very small, and the dataset included repeated trials nested within the same participants; therefore, the reported identification rates should be interpreted as descriptive within-sample results rather than generalizable estimates of classification performance. Second, no independent validation cohort, train/test split, or cross-validation procedure was implemented, limiting conclusions about the algorithm’s robustness and generalizability. Third, because the proposed method is template-based, its performance depends on the representativeness of the reference patterns and on the accurate detection of the start and end of each potential PT. Atypical or compensatory movement strategies, particularly in neurological populations, may therefore affect classification performance. Finally, all acquisitions were performed offline in a controlled laboratory setting with standardized sensor placement and predefined PT tasks. Consequently, the present results cannot be directly extrapolated to real-world environments, where sensor displacement, movement artifacts, partial or interrupted transitions, concurrent activities, and higher signal variability may affect detection and classification. Future studies should include larger and more heterogeneous cohorts, independent validation datasets, clinically relevant motor severity measures, and direct comparison with alternative data-driven approaches.

## 5 Conclusion

This pilot study provides preliminary evidence that a DTW-based algorithm applied to acceleration data from a single sternum-mounted IMU can identify predefined postural-transition signals in an offline dataset, including HS and SwPD. The findings support the technical feasibility of the proposed approach within the present sample, but they should not be interpreted as evidence of validated robustness, clinical effectiveness, or generalizability. Future studies should validate the algorithm in larger and more heterogeneous cohorts, include independent test sets or cross-validation procedures, report participant-level and class-wise performance, compare the DTW-based approach with alternative data-driven classifiers, and assess its performance in online and real-world conditions, such as inpatient rehabilitation settings and home-based or outpatient programs.

## Data Availability

The datasets presented in this study can be found in online repositories. The names of the repository/repositories and accession number(s) can be found below: https://zenodo.org/records/15703161?token=eyJhbGciOiJIUzUxMiJ9.eyJpZCI6ImQ4MDE1MWFhLTJmNzUtNDQ0NC1hMDJjLWFkMjQ4ZTQzYWIyNSIsImRhdGEiOnt9LCJyYW5kb20iOiJhNGRkMDRjZGVlNGM2NGJhODE4NWZjNjNhN2JhNmM0OSJ9.qjyquMCzY2TDydz40-.
